# Exploring the Diversity of Red Microalgae for Exopolysaccharide Production

**DOI:** 10.3390/md20040246

**Published:** 2022-03-31

**Authors:** Aldo Borjas Esqueda, Christine Gardarin, Céline Laroche

**Affiliations:** Clermont Auvergne INP, CNRS, Institut Pascal, Université Clermont Auvergne, F-63000 Clermont-Ferrand, France; aldo.borjas@doctorant.uca.fr (A.B.E.); christine.gardarin@uca.fr (C.G.)

**Keywords:** microalgae, exopolysaccharide, rhodophyta

## Abstract

Microalgae constitute a remarkable biological diversity but a limited number of them have been the object of study for their ability to produce exoplysaccharides (EPS). Among them, the red marine microalgae *Porphyridium* or *Rhodella* produce sulphated EPS, exhibiting some biological activities with potential interest in the pharmaceutical and cosmetic industries. EPS from *Porphyridium* and *Rhodella* being relatively similar in their composition, it has long been considered that all the red microalgae produced similar EPS and no attention was paid to other red microalgae. The objective of our work was then to explore the diversity of red microalgae for the production of EPS, focusing in this first step on the screening of the strains for their ability to produce EPS and preliminary structural characterization. The study was conducted with 11 microalgae strains belonging to the proteorhodophytina subphylum. All microalgae were able to produce EPS, released in the culture medium (strains belonging to Porphyridiophyceae and Rhodellophyceae classes) or remaining bound to the cells (strains from Stylonematophyceae class). The analysis of monosaccharides composition was found significantly different, with for instance high levels of glucuronic acids in the EPS from *C. japonica* and *N. cyanea*, but also strong differences in the sulphation degrees of polymers (between 1.2 and 28.7% eq. SO_4_).

## 1. Introduction

For years, research has been working on the extraction of bioactive molecules offering various application possibilities (food, pharmaceutical, cosmetic). The marine universe, through all its organisms and more particularly algae, offers new sources of bioactive molecules such as polysaccharides, lipids, proteins or pigments. Microalgae are candidates for obtaining new molecules of interest, presenting a great chemical diversity associated with biological activities applicable in different fields such as pharmaceutical or cosmetic. Even if industrial exploitation to obtain molecules of interest is more developed in macroalgae, terrestrial plants, fungi and bacteria, microalgae can find their place as new sources of biomolecules thanks to bioprocess innovations concerning the optimization, reuse and extraction of these molecules, making them competitive in different sectors of activity.

Among those molecules, extracellular polysaccharides (exopolysaccharides, EPS) from microalgae which can easily be extracted from the culture medium, have been described as antiviral, antitumoral, antioxidant, antiparasitic or anti-inflammatory agents [1]. In particular, red microalgae have high EPS production yields. Currently the production of polysaccharides as well as other agri-food products from algal biomass has become attractive for industry but is currently more developed for macro- than microalgae. However, low nutrient and energy requirements make the cultivation of microalgae a sustainable process. In addition, the cost of culture media is low, since their compositions are mainly seawater, nitrates and phosphates, reagent at an affordable price in industrial facilities. Moreover, the use of industrial effluents as culture medium is actually a subject of research interest and could help further decrease of production cost. Nevertheless, the cost of biomass cultivation and processing facilities explains why their industrial development is currently more limited than that of macroalgae that can easily be harvested in the open sea. Among these red microalgae, only a few species have been studied for their ability to produce EPS, while there are about thirty different genera.

Rhodophyta are mainly found between the benthic and intertidal zone, i.e., rather in tropical, temperate waters. It is sometimes found in cold waters. Their development is epiphytic, lithophytic or epibiont. Even if they are marine organisms, some species can be found in fresh water such as rivers, lakes or ponds [2]. The phylum Rhodophyta is a polyphyletic group, containing both microalgae and seaweeds. The classification has been revised several times in the recent years by [3,4]. Depending on the authors, the cyanidiophyceae class is included in the phylum rhodophyta [4], or presented as a different phyla named cyanidiophyte [3]. In the classification of [4], rhodophyta thus include six classes: Bangiophyceae, Compsopogonophyceae, Florideophyceae, Porphyridiophyceae, Rhodellophyceae and Stylonematophyceae. Among these six classes, only the 3 latter contain microalgae, single-celled or mesophilic pseudofilamentous. More recently, this classification has been revised again on basis of plastid genomes sequences [5]. For these authors, the cyanidiophytina is a subphylum of Rhodophyta and the former rhodophytina subphylum has been splitted into 2 different subphylum named proteorhodophytina (containing Compsopogonophyceae, Porphyridiophyceae, Rhodellophyceae and Stylonematophyceae) and Eurhodophytina (Bangiophyceae and Florideophyceae classes). 

**Figure 1:**
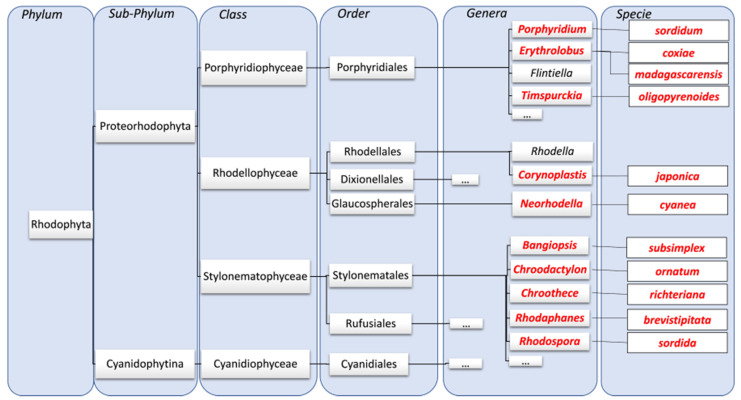
Simplified classification of microalgae in rhodophyta phylum. In red, species included in this study.

Red microalgae for which EPS production has already been described thus belong to the proteorhodophytina subphylum, porphyridiophyceae class (*Porphyridium* and *Flintiella* genus) or Rhodellophyceae class (*Rhodella* and *Dixionella* genus). At this time, only one strain belonging to the stylonematophyceae class (*Chroodactylon ornatum*) has been subject of a study [6], no information being available for the other genus. EPS from *Porphyridium* and *Rhodella* being relatively closed in their structures [7,8], it has long been considered that all the red microalgae produced similar EPS and no attention was paid to other red microalgae. However, a previous work has shown that the red microalga *Flintiella sanguinaria* produce a polymer of different structure, not sulfated, but methylated and acetylated [9]. The objective of our work was then to explore the diversity of red microalgae for the production of EPS, focusing in this first step on the screening of the strains for their ability to produce EPS and preliminary structural characterization. The study has started with 11 microalgae strains belonging to the proteorhodophytina subphylum, and to the 3 different classes: *Erythrolobus madagascarensis, Erythrolobus coxiae, Porphyridium sordidum,* and *Timspurckia oligopyrenoides* (Porphyridiophyceae class), *Neorhodella cyanea* and *Corynoplastis japonica* (Rhodellophyceae class), *Chroodactylon ornatum, Chroothece richteriana, Bangiopsis subsimplex, Rhodospora sordida*, and *Rhodaphanes brevistipitata* (Stylonematophyceae class). Figure 1 presents a simplified classification of the studied strains among Rhodophyta phylum. To achieve our objectives, methodology used in this study was first to highlight potential presence of EPS using several staining methods and microscopic observations. As a second step those strains have been cultivated in standardized conditions (same medium, irradiance and temperature for all). Growth and EPS production were evaluated, and EPS were further extracted and characterized using colorimetric assays, infrared spectroscopy and monosaccharide analysis.

## 2. Results

### 2.1. Microscopic Observations and Staining

**Figure 2:**
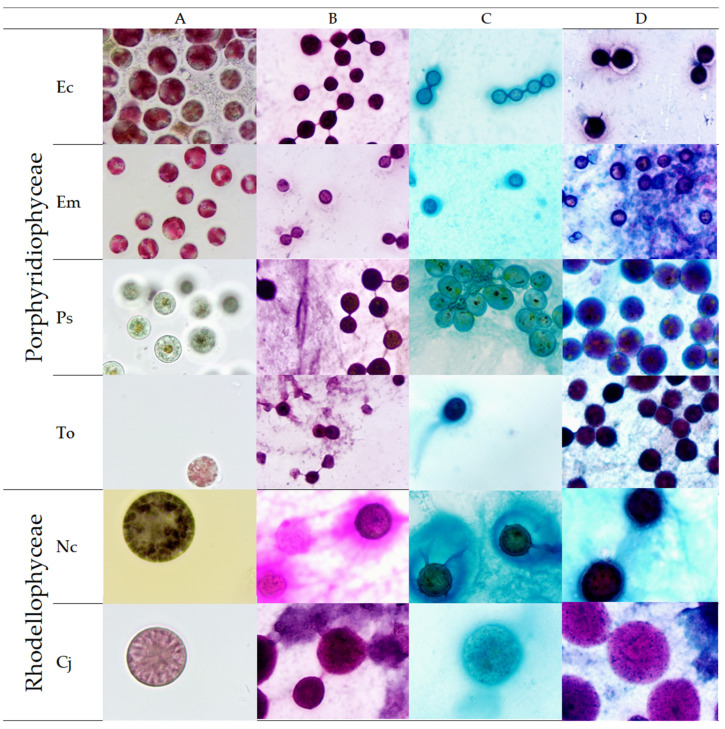
Microscopic observations of porphyridiophyceae and rhodellophyceae strains, before (**A**) and after staining with Schiff/periodic acid (**B**), alcian blue (**C**) and combined staining (**D**). Ec: *Erythrolobus coxiae*, Em: *Erythrolobus madagascarensis*, Ps: *Porphyridium sordidum*, To: *Timspurckia oligopyrenoides*, Nc: *Neorhodella cyanea*, Cj: *Corynoplastis japonica*.

Microalgae strains were first observed under bright-field microscope without staining (Figure 2A). For *E. coxiae, E. madagascarensis, T. oligopyrenoides* and *P. sordidum*, cells appeared as unicellular and spheric shape, with closed mean diameters around 8–10 µm. The main difference concerned the green-olive color of *P. sordidum* cells, whereas other strains were found brownish-red due to the highly probable presence of phycoerythrin. These observations are in accordance with the papers describing ultrastructure of these strains [10,11,12]. For rhodellophyceae strains (*N. cyanea* and *C. japonica*), the mean diameters were found much greaters (~40 and ~35 µm, respectively), indicating more variability of this parameter for strains belonging to this class, as *Rhodella violacea* or *Rhodella maculata* sizes are described to be around 8–10 µm [13]. However, it is to notice that according to the last classification, *Neorhodella cyanea* belongs to the glaucospherales order, while *Corynoplastis* japonica is found within the rhodellales order. The color of these 2 strains is also different, with a light pink color for *C. japonica*, and dark grey/olive-green color for *N. cyanea*. These 2 strains have been previously described for their ultrastructure, and our observations are in accordance with these papers [14,15]. After this first microscopic observation, different staining protocols were applied in order to visualize presence or absence of polysaccharides and to give a first approach on their composition. Indeed, the first staining was performed using the periodic acid-Schiff reagent (PAS). Periodic Acid react with vicinal diols of polysaccharides to form aldehyde groups, while Schiff reagent will further bind them, leading to a pink to purple color. Hematoxylin counterstain can also be used to demonstrate cell nuclei in blue. As a result, all polysaccharides (intra or extracellular) will be stained. This method is classically used in histology to highlight presence of glycogen, glycoproteins and mucopolysaccharides in tissues, but has been successfully transposed to our microalgae samples by adaptation of protocol. The second staining method used Alcian Blue, which is a cationic compound and will bind specifically to negative charges of polysaccharides, allowing to visualize carboxylated and/or sulphated polysaccharides. Finally, a “combined staining” protocol was applied, allowing to distinguish neutral polysaccharides (that not bind to Alcian Blue, thus remaining pink/purple) from carboxylated and/or sulphated polysaccharides (appearing in blue). 

For Porphyridiophyceae strains (*T. oligopyrenoides, P. sordidum, E. coxie* and *E. madagascarensis*), cells stained with PAS reagent (Figure 2B) appeared with intense pink/purple color, with sharp edges and kind of “links” between cells. Moreover, all the slide was with a slight pink color, indicating presence of soluble polysaccharide in the medium. For Rhodellophyceae strains (*N. cyanea* and *C. japonica*), same kind of observations are made, but with more diffusion of EPS from cells to medium. With Alcian Blue staining (Figure 2C), these soluble polysaccharides appeared in light blue, while the staining of cells was more intense. However, blurred edges can show some diffusion of the PS from the cells to the medium, with larger dark blue zone around *N. cyanea* cells suggesting a larger release of EPS for this strain. It has been shown that *Porphyridium* strains, such as *purpureum/cruentum* produce large amounts of EPS, part of it remaining tightly bound to the cells (typically called Bound Polysaccharide BPS or capsule CPS or sheath), while another part is released to the medium as soluble polysaccharide (sometimes called Released Polysaccharide, RPS). For these strains around 50 to 70% of polysaccharide could be found as BPS [16], but this amount could vary depending on the culture conditions. Our observations suggest thus that the 4 porphyridiophyceae and the 2 Rhodellophyceae strains included in this study could have the same comportment as PS are present both surrounding the cells and in the medium. With the combined staining PAS/AB (Figure 2D), an intense blue color of the cells is observed, with for some strains (*E. coxiae, E. madagascarensis, P. sordidum* and *T. oligopyrenoides*) some pink/red color visible inside cells, showing presence of neutral intracellular polysaccharide that may be starch. For *N. cyanea*, this neutral PS is more hardly seen, suggesting a lower amount of starch accumulated in our culture conditions, or a masking of it due to the dark blue AB staining. Finally, for *C. japonica*, cell’s surface seemed not to be uniformly covered by the anionic polysaccharide, as it was possible on some cells to see a pink coloration but with blue points on the cells’ surface. 

**Figure 3:**
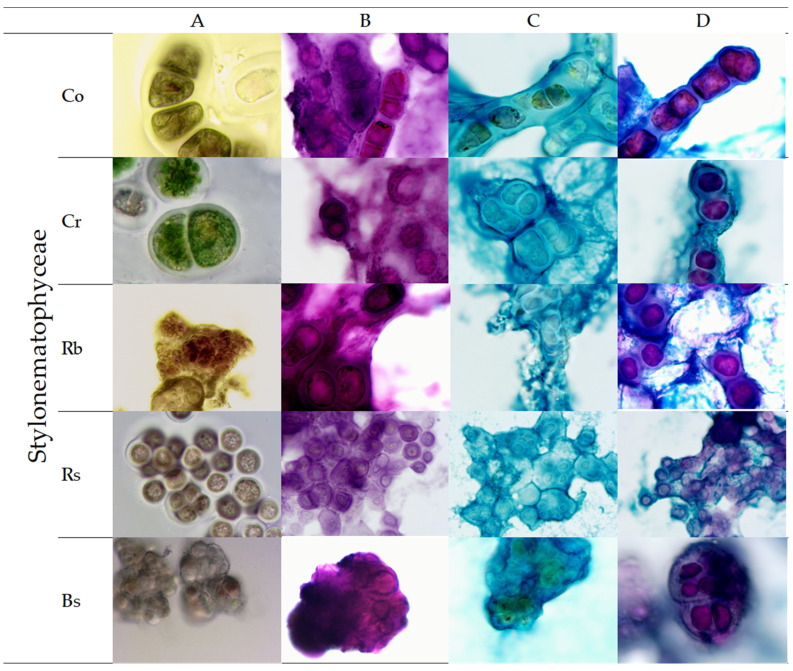
Microscopic observations of stylonematophyceae strains, before (**A**) and after staining with Schiff/periodic acid (**B**), alcian blue (**C**) and combined staining (**D**). Co: *Chroodactylum ornatum*, Cr: *Chroothece richteriana*, Rb: *Rhodaphanes brevistipitata*, Rs: *Rhodospora sordida*, Bs: *Bangiopsis subsimplex*.

The Stylonematophyceae class is quite large. It mainly contains filamentous, pseudo-filamentous or multicellular aggregates (such as *Bangiopsis, Chroodactylon, Chroothece, Kyliniella* and *Stylonema)*, but some unicellular microalgae are also encountered such as *Rhodosorus, Rhodospora* et *Rufusia* [17]. For Stylonematophyceae strains without staining (Figure 3A), strains appeared thus with different morphologies. *C. richteriana* cells are described as ellipsoid or cylindrical (~12 µm width and 15 µm length) surrounded by a mucilaginous envelope that may grow unilaterally. This can develop into a stalk, connected with the basal part of the sheath surrounding cells [18,19]. Depending on the culture conditions, *C. richteriana* cells can develop as free-living cells and colonies or under pseudo-filamentous form [18]. In the present study, cells were only observed under small colonies and no stack was noticed. *Rhodaphanes brevistipitata* morphology has been described as a short thallus (<1.1 mm) uniseriate to multiseriate (2 to 10 cells wide). Reproduction is observed by release of individual vegetative cells, or when the whole thallus gelatinizes and releases a mass of free cells [20]. Our observations are thus in accordance with this description. *Chroodactylon ornatum,* as previously described by [2], appeared as pseudo-filamentous, with cells size around 10 µm large and 15–20 µm long, and filaments that can be as long as 1–2 mm. For *B. subsimplex*, [21] has described a small thallus composed of a flexible filament, gelatinous, up to 1.5 mm long, with cells wrapped in individual sheaths, spaced one from the other by a common gelatinous mass. In the present study only these individual cells and gelatinous mass have been observed. Finally, *Rhodospora sordida* cells were observed as single cells around 8 µm diameter and sometimes as 2 to 4 cells surrounded by a thick and clear capsule. These observations were consistent with previous study [22]. Thus, for *C. ornatum, C. richteriana* and *R. sordida*, a clear capsule was observed around cells, while *B. subsimplex* and *R. brevistipitata* appeared as cells aggregates, without evidence for an external polysaccharide layer but more a gelatinous mass surrounding cells. After PAS staining (Figure 3B) some polysaccharidic matrix surrounding cells appears as evidence. These capsules and polysaccharidic matrixes are stained by the AB method (Figure 3C), showing that they are carboxylated and/or sulphated. However, intensity of staining is lower than for Porphyridiophyceae and Rhodellophyceae strains and no clear coloration of surrounding medium is observed, suggesting that PS remain bound to the cells and are not released to the medium. Additionally, chlorophyll is still visible inside cells for *C. ornatum, C. richteriana* and *B. subsimplex*, suggesting that these PS are only found extracellularly. This is confirmed by the combined PAS-AB staining (Figure 3D), with some neutral intracellular polysaccharides strongly stained in purple to red, and acidic/sulphated PS outside cells. Interestingly, for *C. ornatum*, 2 different colors are observed inside cells, with purple quite evenly distributed and some red spots that may be supposed to be starch granules, suggesting thus presence of 2 different PS inside cells.

### 2.2. Growth and Exopolysaccharide Production

**Figure 4:**
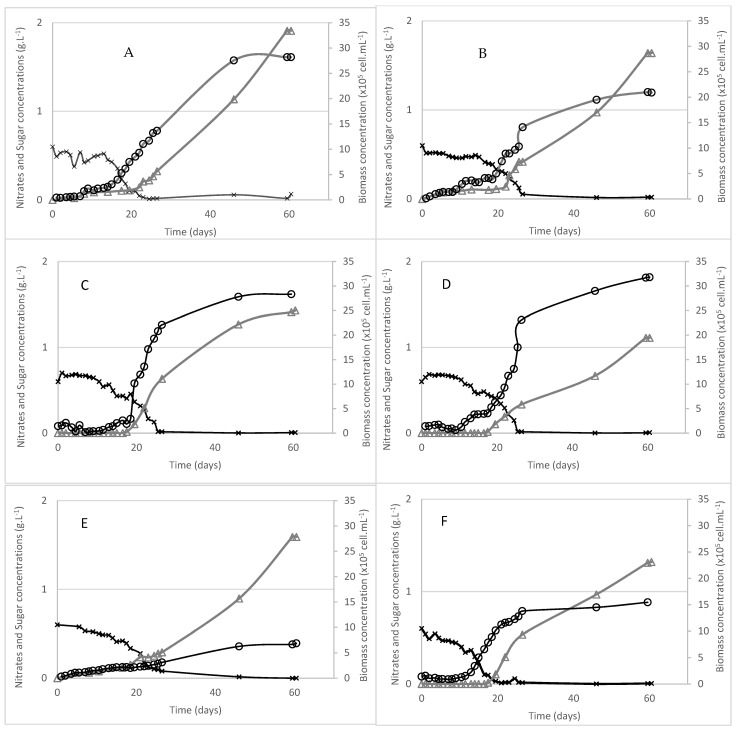
Biomass (open circles), nitrates (crosses) and extracellular sugar concentrations (triangles). (**A**) *Porphyridium sordidum*; (**B**) *Erythrolobus madagascarensis*; (**C**) *Erythrolobus coxiae*; (**D**) *Timspurckia oligopyrenoides*; (**E**) *Corynoplastis japonica*; (**F**) *Neorhodella cyanea*.

Due to the filamentous or pseudo-filamentous comportment of stylomenatophyceae strains, it was not possible to follow growth by counting cells under microscope. Moreover, biomass aggregates made impossible the biomass evaluation during the time course of the growth, even by dry weight measurements, as the sampling would not have been accurate due to heterogeneity of the culture. Thus, only final biomass was determined for these strains (*Chroodactylon ornatum*, *Bangiopsis subsimplex*, *Chroothece richteriana*, *Rhodaphanes brevistipitata*, and *Rhodospora sordida)*. For porphyridiophyceae and rhodellophyceae strains (*Porphyridium sordidum*, *Erythrolobus madagascarensis*, *Erythrolobus coxiae*, *Timspurckia oligopyrenoides*, *Corynoplastis japonica* and *Neorhodella cyanea)*, growth, nitrate consumption, and exocellular sugar production kinetics have been reported on Figure 4. 

For all strains, profiles are quite similar, with a growth associated with nitrate consumption. However, significant differences are observed in terms of growth rates (from 0.067 d^−1^ for *C. japonica*, to 0.263 d^−1^ for *E. coxiae*) leading to inverse order doubling time, from 2.63 days for *E. coxiae* to 10.34 days for *C. japonica*. In studies on other red microalgae strains, apparent µmax have been estimated to 0.235 d^−1^ for *Flintiella sanguinaria* [9], 0.38 d^−1^ for *Porphyridium cruentum* [23], or 0.79 d^−1^ for *Porphyridium marinum* [7]. Recently, a first study on *Porphyridium sordidum* and its comparison with *Porphyridium purpureum* has shown apparent µmax at 0.17 and 0.22 d^−1^, respectively [24]. However, it is very difficult to compare from one study to another as these parameters are not always calculated, and as it is strongly dependent on culture conditions such as salinity [9] and light availability (linked to the provided irradiance but also on the design of culture vessel and thus on light diffusion inside culture). 

Nutrient limitation has been described as an inducing factor in the production of EPS. When the amount of nitrogen decreases, growth stops resulting in the beginning of the stationary phase. However, microalgae still have the ability to perform photosynthesis and continuous carbon fixation. During this step, excess carbon is redirected to the formation of energy reserves such as starch and lipids, or excreted in the form of EPS [25]. For all strains for which it was possible to follow the growth, EPS synthesis was at its maximum when nitrogen was in limitation or depletion condition. Nevertheless, for two strains (*C. japonica* and *P. sordidum*), entry in stationary phase is not observed immediately after nitrogen depletion, and EPS synthesis seems then to occur while cells are still in linear growth phase. Induction of EPS synthesis by nitrogen deprivation has previously been described for several species of red microalgae, such as *Porphyridium* sp. [26], *Porphyridium marinum* [7], *Rhodella violacea* [8] or *Flintiella sanguinaria* [9]. This behavior is therefore not surprising. Nevertheless, it would be interesting to test other stresses described to induce the synthesis of EPS, such as phosphorus deficiency or light stress, in order to identify the operating condition allowing the best production.

The growth kinetics of Stylonematophyceae strains could not be established due to the filamentous appearance of the cells. Nevertheless, the visual aspect of the cultures shows that the biomass has developed. This is confirmed by monitoring the nitrate concentration, even if the kinetics of consumption seem slower than for other species.

At the end of the cultivations period, biomass was harvested by centrifugation, and supernatant was diafiltered to recover EPS. The biomass (in g L^−1^ and number of cells mL^−1^) and EPS concentrations (g L^−1^) obtained at the end of cultivation, as well as the production yields of EPS (g EPS/g DW biomass and g EPS/10^6^ cells) are presented in Table 1.

Final biomass concentrations obtained for Porphyridiophyceae and Rhodellophyceae were not very different from one strain to the other, from 1.79 g L^−1^ for *E. madagascarensis* to 2.49 g L^−1^ for *C. japonica*. However, strong differences are noticed regarding number of cells, from 6 × 10^6^ cells mL^−1^ for *C. japonica*, 15.4 × 10^6^ cells mL^−1^ for *N. cyanea*, to 31.8 × 10^6^ cells mL^−1^ for *T. oligopyrenoides*. These differences can be explained by cells diameter. Indeed, correlations established between dry weight and number of cells were in the order of 0.65 mg DW/10^6^ cells for *P. sordidum, E. madagascarensis, E. coxiae* and *T. oligopyrenoides* which have a mean diameter of 8–10 µm, whereas they were about 0.152 mgDW/10^6^ cells for *N. cyanea* (diameter of ~40 µm) and 0.415 mg DW/10^6^ cells for *C. japonica* (diameter of ~35 µm).

Regarding EPS production, final concentrations were found between 1.12 g L^−1^ for *T. oligopyrenoides*, to 2.32 g L^−1^ for *N. cyanea*. Productivities can be calculated as a DW basis (g EPS/g DW biomass), or as a cell basis (mg EPS/10^6^ cells), leading to different conclusions.

On a dry weight basis, productivities were found between 0.35 (*T. oligopyrenoides*) and 0.99 g EPS/g DW biomass (*N. cyanea*). However, on a cell basis, the best EPS producer appears to be *C. japonica* with 0.265 mg EPS/10^6^ cells. These differences can be attributed to the difference in size and weight of cells as previously discussed. Rhodophyta are described to be among the “best producers” of EPS. Most microalgae from other phyla described to produce EPS are able to achieve concentrations at maximum between 0.5 and 1 g L^−1^ [27]. On the other hand, the optimization of EPS production by *P. marinum* made possible to reach a final concentration of 4.1 g L^−1^ (productivity of 0.067 mg/10^6^ cells), while it was 1 g L^−1^ before optimization [7]. For *F. sanguinaria*, optimization of culture conditions led to a final concentration of 1.2 g L^−1^ and productivity of 0.13 mg/10^6^ cells [9]. Even if the standardized conditions used during this study were not optimized, the concentrations and productivities obtained in this study are thus already far from insignificant. Indeed, the maximum productivity in EPS varies according to the species, and a case-by-case optimization should be run in order to improve the production, but these first results are thus really promising in terms of production yields. All microalgae strains don’t react in the same way to nitrogen deprivation, some of them redirecting the excess carbon flux toward lipids or starch accumulation instead of EPS synthesis, and other kind of stress (phosphorus deficiency, light) would be interesting to be tested to further improve EPS production yields. Moreover, [28] described the formation of 2 types of EPS depending on nutrient status; the first type was produced under non-limiting conditions and the second under limitation, revealing different mechanisms implicated in carbon excretion, some of which are not necessarily linked to metabolic overflow processes.

Concerning strains from Stylonematophyceae class, biomass concentrations reached were lower, about 0.46, 0.51, 0.64, 0.72, and 1.2 g L^−1^ for *B. subsimplex, R. brevistipitata, C. richteriana, R. sordida* and *C. ornatum*, respectively. *C. ornatum* is a freshwater microalgae that can nevertheless grow in saline waters [2]. Even in a marine medium such as F/2 medium, *C. ornatum* has thus developed significantly, but the high salinity may have slowed down growth. Apart from salinity, other cultivation parameters such as light or temperature could have limited growth and biomass concentration should probably be improved. Moreover, despite the significant growth, the exopolysaccharides released in the culture medium were found negligible for all Stylonematophyceae strains since almost no extracellular sugar were detected during cultivation, and only 0.011 to 0.023 g L^−1^ of EPS were recovered after purification. As seen with cells staining, these strains produce a carboxylated and/or sulphated polysaccharide, but which remains probably tightly bound to the cells under the form of a capsule. Thus, since polysaccharides are not free in the culture medium, they cannot be quantified and extracted by simple centrifugation. In order to recover these polysaccharides, a specific protocol was used, modified from [29]. This protocol has been developed specifically to extract acidic BPS preventing contamination of the extracts with neutral intracellular polysaccharides. Productivities calculated are thus comprised between 0.08 (*B. subsimplex*) and 0.24 g BPS/g DW biomass (*C. ornatum*). Even if these productivities are found lower than for Porphyridiophyceae and Rhodellophyceae strains, they are not negligible, and could probably be further improved.

### 2.3. Exopolysaccharide Characterization

**Figure 5:**
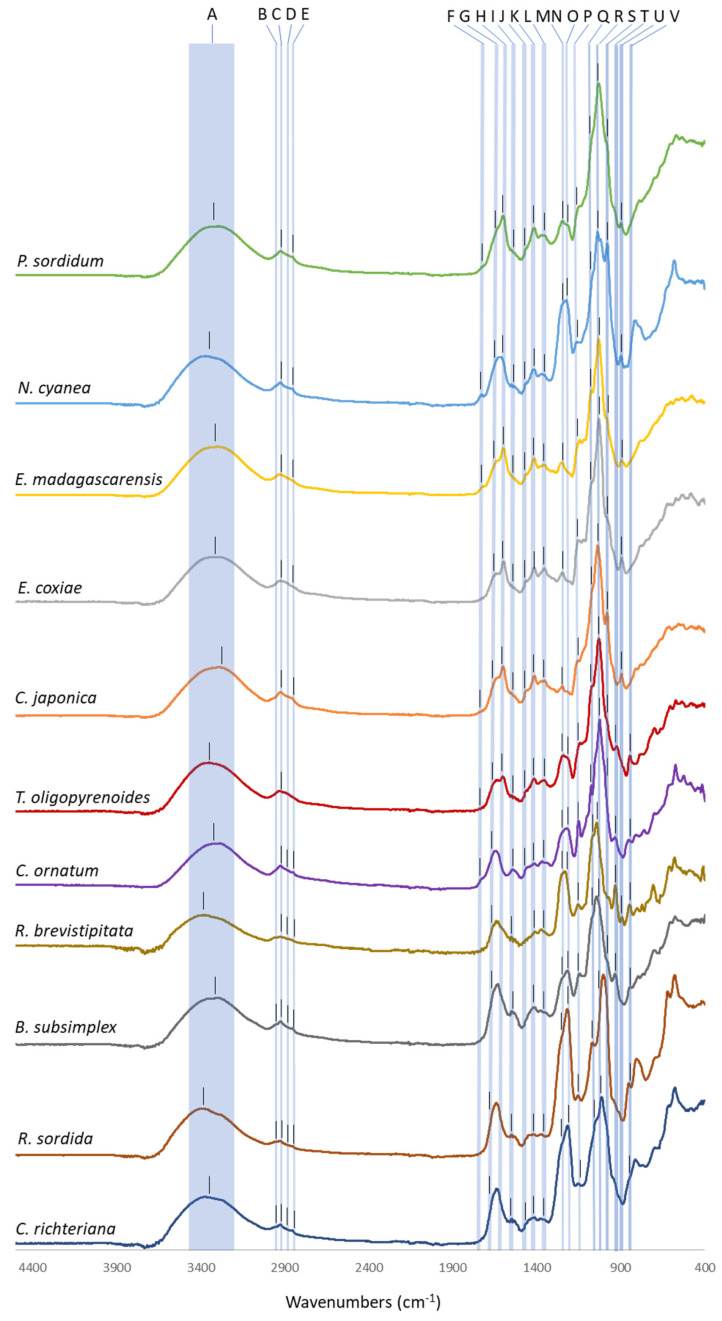
FT-IR spectra of EPS. Characteristic bands (labelled from A to V) refer to assignments indicated in Table 2.

The Fourier Transform InfraRed (FT-IR) spectra of EPS extracts (RPS or BPS), recorded between 5000 and 400 cm^−1^, are presented on Figure 5, showing some typical bands of polysaccharides, as well as more specific peaks relied to the presence of substituents on the polymer’s backbones. Characteristic bands (labelled from A to V) refer to assignments indicated in Table 2. Indeed, the broad band around 3300–3350 cm^−1^ was assigned to hydrogen bonded O-H stretching vibrations and two others at 2923 and 2853 cm^−1^ can be attributed to symmetric and asymmetric stretching vibrations of CH_2_ groups [30]. Typical bands from polysaccharides were found between 1200 and 950 cm^−1^ [31], corresponding to stretching vibrations of pyran rings (C-O-C, C-OH and C-C), identified, respectively, around 1145, 1075 and 1030 cm^−1^ for all samples.

Among specific bands, carboxylate can be identified by presence of symmetric and asymmetric stretching vibrations of deprotonated carboxylic function (COO^−^) at 1585–1605 cm^−1^ and 1400–1420 cm^−1^ [32,33,34]. For all strains peaks were detected around 1413 cm^−1^, showing that all EPS are carboxylated. However, only EPS from Porphyridiophyceae and Rhodellophyceae have shown the second one around 1600 cm^−1^. This can probably be explained by a lower uronic acids level for EPS from Stylonematophyceae strains, and this band can be masked by another peak. Presence of sulphate groups can be observed by bands at 1215–1225 cm^−1^, and 1245–1255 cm^−1^ [35,36,37]. For all EPS analyzed during this study, peaks were detected in this latter range, however, vibrations of *O*-Acetyl groups are generally seen in the range 1240–1250 cm^−1^, leading to difficulties to assign bands recorded at these wavenumbers. In the range 1215–1225 cm^−1^, peaks were assigned for EPS from *T. oligopyrenoides, N. cyanea, P. sordidum, C. ornatum, R. brevistipitata, B. subsimplex, C. richteriana* and *R. sordida*. In some cases (EPS from Stylonematophyceae and *N. cyanea*), this bands were even found of high intensity, suggesting a high sulphation degree. At the opposite, it was not possible to detect it for *C. japonica, E. madagascarensis* and *E. coxiae*, showing that sulphate groups can be absent or at low level in these EPS. EPS from *Porphyridium purpureum, cruentum, marinum* and *Rhodella violacea* are sulfated, while the one from *Flintiella sanguinaria* wears only low sulphate groups, but methyl and acetyl groups on its backbone [9]. Apart from the band at 1240–1250 cm^−1^, *O*-Acetyl groups can be seen in the range 1725–1740 cm^−1^ [38]. On our recorded spectra, this vibration was identified only for *T. oligopyrenoides, E. madagascarensis, C. japonica,* and *N. cyanea,* suggesting that only these EPS can present *O*-acetyl groups on their backbone. Symmetric and asymmetric stretching vibrations of methyl groups are generally encountered at 2950–2960 and 2870–2880 cm^−1^, respectively, whereas CH_3_ bending vibration occurs at 1455–1470 cm^−1^. If this latter one can be clearly seen for Porphyridiophyceae and Rhodellophyceae strains, it was not the case for Stylonematophyceae ones suggesting a low or no methyl pattern. If methylated sugars are rather frequently found in polysaccharides from seaweeds, their occurrence is less described in EPS from microalgae, probably due to a lack of in-depth characterization. Nevertheless, their occurrence may be much more frequent than currently described. Identification of the position of these methyl groups is even more rare mainly due to a lack of standards. However, some studies have shown presence of 3-*O*-Me-xylose, 4-*O*-Me-xylose, 2,3-di-*O*-Me-rhamnose and 2,3-di-*O*-Me-fucose in the composition of EPS from the red microalgae *Rhodella grisea* [39], whereas di- *O*-Me-hexose, 4-*O*-Me-galactose [40] and 6-*O*-Me-mannose [41] have been detected in EPS of *Porphyridium* sp. Methyl groups can also be found on uronic acids, as 2-*O*-Me-glucuronic acid was highlighted on EPS from *P. cruentum* [42]. Moreover, presence of acetylated and/or methylated glucuronic acid has been suspected in the EPS from *F. sanguinaria* [9]. *O*-acetyl groups are poorly described on EPS from photosynthetic microorganisms, except for cyanobacteria such as *Cyanothece* [43] or *Nostoc* strains [44] but analysis of EPS from *Rhodella grisea* has suggested their presence, but no quantification was performed [39]. 

A band around 930 cm^−1^ has been detected for *C. ornatum, R. brevistipitata, B. subsimplex*, and *T. oligopyrenoides*. A peak at this wavenumber is often encountered for polysaccharides from seaweeds and is attributed to C-O-C vibrations of 3,6-anhydrogalactose [45]. It is thus a little bit surprising, as to our knowledge, this is the first time anhydrogalactose is suspected to enter in the composition of an EPS from microalgae. However, for strains from Stylonematophyceae, it seems credible that EPS contains anhydrogalactose, as they exhibit a filamentous or pseudofilamentous morphology, quite intermediate between unicellular microalgae and macroalgae, so their EPS may also have an intermediate structure. For *T. oligopyrenoides*, it is even more surprising, but the peak is seen at 923 cm^−1^ so not exactly in the range described in literature (925–935 cm^−1^) and could perhaps correspond to another vibration. In all cases, further experiments will be needed to confirm (or deny) presence of these 3,6 anhydrogalactose in our EPS.

On IR spectra, some peaks can indicate the main glycosidic linkage in the polymer, as peak at 890–900 cm^−1^ corresponds to a β anomeric configuration of C_1_ (β[1-?]), while 840–850 cm^−1^ corresponds to α-linkage of C_1_ (α[1-?]) [46]. In our study, spectra recorded for EPS from *E. coxie, E. madagascarensis, P. sordidum, C. japonica* and *N. cyanea* show that the main glycosidic bond in these polymers should be of β[1-?] type. This was also the case in a previous study on the EPS of *F. sanguinaria* [9]. However, it can’t be considered as a general feature of EPS from Porphyridiophyceae strains, as for *T. oligopyrenoides*, the main linkage seems surprisingly to be of α[1-?] type. For Stylonematophyceae strains, all FT-IR spectra have shown peaks corresponding to α linkage, but for *R. brevistipitata*, a peak for β linkage is also seen suggesting a mix of both glycosidic bond types for this strain. 

Finally, for all spectra, peaks at 1525–1540 cm^−1^ could be assigned to amide II band of proteins, suggesting their presence in our samples.

To go further into EPS characterization, global composition has been evaluated by colorimetric assays, in order to evaluate total sugars (purity of the extracts), neutral sugar, uronic acids, proteins and sulphate groups contents (Table 3).

Total sugar assay has shown a slightly lower purity of EPS extracted from the Stylonematophyceae class, which is not surprisingly due to the much more complex extraction method. Considering this purity, results of neutral sugars, uronic acids and sulphates have been expressed as % mass in the EPS. Neutral sugar content was found between 61 and 80% for all strains, except for *N. cyanea* were less than 40% neutral sugars were detected. Concerning uronic acids, EPS from Porphyridiophyceae and Rhodellophyceae strains present a greater content (from 18 to 37%) than stylonematophyceae ones (from 2 to 12%). These values are globally in agreement with previous studies on other red microalgae, as 20% were described for EPS from *F. sanguinaria* [9], 18% for *P. purpureum* [48], 17.5% for *P. marinum* [7] or 9% for *R. violacea* [8]. In the present study, four EPS were found to have more than 30% uronic acids (*E. coxiae, E. madagascarensis, C. japonica* and *N. cyanea*) that represent a noticeable amount. However, colorimetric assays results should be taken with care, as the accuracy of the method is not absolute. Only a few species of microalgae and cyanobacteria have been described to contain >20% levels of uronic acids, including *Exanthemacrysis* sp., *Nostoc insulare, Chlamydomonas agustae, Closterium* sp. and *Amphora* sp., for which levels of 37, 26, 28, 35 and 55%, respectively, were detected [49,50,51,52,53].

Concerning sulphation degree, it was found really variable between samples. EPS from Stylonematophyceae strains arbour a high sulphate content, from 18% for *R. brevistipitata* to ~29% for *R. sordida*. However, for Porphyridiophyceae and Rhodellophyceae strains, sulphate content was found much more variable, with low sulphation degree for *C. japonica, E. coxiae and E. madagascarensis* (1.2, 1.6 and 1.9%, respectively), whereas the one from *N. cyanea* appears sulphated up to 24%. 

This variability has also been noticed for EPS described in previous studies, as EPS from other red microalgae are generally sulphated between 4 and 10%, as shown on *Porphyridium* sp. (8.5%, [54]), *P. marinum* (10.4%, [7]) or *R. violacea* (4.7%, [8]). However, analysis of EPS from *F. sanguinaria* had shown presence of a non-sulphated polymer [9]. Recently, [24] found 13% sulphate groups for EPS from *P. purpureum*, and 18% for *P. sordidum*. Our value of 6.8% for EPS from *P. sordidum* appears thus much lower than in this previous study. However, culture conditions and method for sulphate content evaluation were different and could then explain such different results. As biological activities of polysaccharides are often attributed to the presence of negative charges, EPS from *N. cyanea* appears then really promising at this step, as this polymer exhibits both high uronic acid (37%) and sulphate (24%) contents.

Finally, proteins have been detected in all samples, between 5 to 12% depending on EPS.This result is in accordance with other studies, as proteins are often described for EPS from microalgae [27]. For EPS from red microalgae, this content was found around 9% for *Porphyridium marinum* [7], 5% for *Flintiella sanguinaria* [9], and up to 16% for *Rhodella violacea* [55]. This high protein content has led to the use of proteoglycan for the polymer produced by some species of the *Rhodella* genus. However, existence of a covalent linkage between protein and glycan parts has never been demonstrated, and their presence can be due to the extraction and purification methods.

Monosaccharides composition was evaluated by HPAEC-PAD after acidic hydrolysis and expressed as molar ratio of the identified peaks. Results are presented in Table 4, Table 5 and Table 6 (Table 4 Porphyridiophyceae, Table 5 Rhodellophyceae, Table 6 Stylonematophyceae), and chromatograms of standards and EPS samples are proposed as supplementary material (Figure S1). Microalgae have the ability to produce complex polymers whose osidic composition varies up to eight monosaccharides. Most abundant monosaccharides are glucose, galactose and fucose and in some cases rhamnose and arabinose [27,56,57]. Nevertheless, these compositions are very variable depending on the phyla belonging to the microalgae. Thus, [27] showed that one of the characteristics of red microalgae EPS could be the significant presence of xylose, which is often the main monosaccharide in these polymers, the 2nd most represented monosaccharide being galactose. However, some authors described the reverse order (majority of galactose and secondary xylose). In addition, all red microalgae EPS analyzed to date contained a significant amount of glucuronic acid [27]. Nevertheless, these previous studies concerned only few red microalgae strains. Moreover, the differences observed by some authors can be explained by different cultures conditions, but also by the method used for extraction and quantification. Indeed, [58] has shown that the monosaccharide composition of microalgae EPS is strongly dependent on these methodologies.

The main monosaccharide present in the Porphyridiophyceae strains analyzed is xylose (from 46 to 56% molar), followed by galactose (from 12 to 28%), and glucuronic acid (from 18 to 21%). Some other monosaccharides are nevertheless found in various amounts in these EPS, such as glucose (from 1.0 to 12.8%), fucose (up to 4.5% for *E. madagascarensis*), but also traces of arabinose, rhamnose or galacturonic acids. From a general point of view, the composition of polysaccharides extracted from most microalgae and cyanobacteria show the presence of negative charges thanks to uronic acids, but also due to the presence of sulphate groups. The presence of uronic acids for all EPS analyzed is therefore not surprising, and levels of 18% (for *P. sordidum*) to 21% (for *E. madagascarensis* and *E. coxiae*) are in accordance with what is usually found in the literature for Porphyridiophyceae. In the recent study of [24], EPS from *Porphyridium sordidum* was found to contain 39% xylose, 33% galactose, 23% glucose, and 5% glucuronic acid, with 18% of sulphate groups (molar % have been recalculated from mass % data available in the paper to compare with our study). The composition obtained in our study is a little bit different, with 46% xylose, 21% galactose, 13% glucose, and 18% glucuronic acid, with 6.8% of sulphate groups, thus showing greater uronic acids and less sulphate groups. However, as previously mentioned, the compositions can vary both with culture conditions and with the methodology used for extraction and quantification [58].

For Rhodellophyceae, EPS compositions were found more surprising as EPS described until now in the literature for *R. violacea* and *R. maculata* described galactose and xylose as the main monosaccharides [7,48]. For *C. japonica,* xylose is the main monosaccharide (50%), but the 2nd one being glucuronic acid with a high content of 26%, and the 3rd one rhamnose (14%), galactose being quantified only at 9% in the polymer. The other strain of Rhodellophyceae (*N. cyanea*) also contains glucuronic acid as the 2nd most abundant monosaccharide (25%), but this time with a low rhamnose content, and as for *C. japonica,* only 10% of galactose. These low galactose amounts, and high glucuronic acids thus set them apart from other Rhodellophyceae species, as only 3 to 5% were detected in EPS from *R. violacea* and *Rhodella maculata*, respectively [7,48].

Finally, these preliminary compositions do not take into account some peaks of very significant areas that remain unidentified due to a lack of standards. This type of peak can appear when *O*-methylated monosaccharides enter in the composition of the polymer, as demonstrated in *F. sanguinaria* [9]. In the case of *F. sanguinaria*, analysis of native and desubstituted EPS revealed that methylated monosaccharides are probably glucose and glucuronic acid. In the present study, a peak was observed at the same retention volume for all EPS from Porphyridiophyceae and Rhodellophyceae strains, suggesting that they can contain methylated uronic acid. This finding can be related to the observations made on FT-IR spectra, as presence of methyl groups is suspected for the same strains. The application of a desubstitution protocol will therefore be necessary, both to quantify the methyl and acetyl groups, but also to re-analyze the composition in monosaccharides and access more reliable quantifications. 

Regarding BPS from Stylonematophyceae strains, a certain variability in compositions is observed. However, the general tendency is galactose, xylose and glucose as the 3 main monosaccharides, thus confirming the high neutral sugar amounts detected by colorimetric assay. Moreover, low amounts of uronic acids were detected in these samples, this result being confirmed by the HPAEC-PAD analyze as they contained only between 2% (*R. brevistipitata*) to 12% (*C. ornatum*) of glucuronic and galacturonic acids. However, the composition of the EPS from *C. ornatum* is not in accordance with the previous study [6]. Indeed, these authors proposed a structure of sulphated galactan type, close to what is generally observed in red seaweeds. However, extraction protocol used in their study is really different from the one described in the present paper, and similar to what is generally used to extract cell wall and matrix polysaccharides from seaweed, such as cellular lysis, hot water extraction and alcoholic precipitation. Here, the methodology used is specifically directed towards anionic polysaccharides of capsules, and no cellular lysis was observed (data not shown). Thus, *C. ornatum* may contains several polysaccharides, with one or several neutral ones, and an anionic extracellular one. This hypothesis is also supported by the combined staining observations, for which high proportion of neutral PS were observed inside cells.

## 3. Discussion

The present paper is thus the first study aiming to explore the diversity of red microalgae for their ability to produce EPS. One of the main findings was the high production yields observed for Porphyridiophyceae and Rhodellophyceae strains (even if the culture conditions were not optimized), and the fact that the strains belonging to the Stylonematophyceae class exhibit only bound exopolysaccharides. Regarding the compositions, some common features were highlighted, such as presence of xylose, galactose, glucose and glucuronic acid in all compositions, but with different amounts depending on samples. Moreover, presence of sulphate groups was found much more variable. 

The EPS produced by microalgae and cyanobacteria have been the subject of numerous publications regarding their biological activities such as antioxidant, antiviral, antifungal, antibacterial, anti-ageing, anticancer and immunomodulatory agent [1].

Regarding specifically EPS from red microalgae, antioxidant activities were highlighted, with a dose-dependent activity, for *Porphyridium* sp., *P. cruentum*, and *Rhodella reticulata* [59,60,61,62]. The sulfated EPS from *Porphyridium* sp. has shown an antiviral effect, against the herpes virus (HSV-1 and HSV-2; [63,64]). Antibacterial and antifungal properties have also been described, with strong variations in minimum inhibitory concentrations (MIC) from a polymer to another, and from a bacterial strain to another as shown for EPS from *P. marinum* [65]. Some antitumor activities have also been highlighted [65,66,67] and in most cases, the activity has been related to the immunomodulatory effect of polymers. Indeed, the EPS from *Porphyridium cruentum* improves the immune response by stimulating the proliferation of macrophages and lymphocytes. The EPS from microalgae can also be used as anti-inflammatory agents. This is the case of the polymer isolated from *Porphyridium* sp. which inhibits the migration of leukocytes to the site of inflammation [68]. 

Despite all these studies demonstrating biological activities of EPS from red microalgae, mechanisms by which they act remains almost unclear and it is generally difficult to link an activity to a polymer structure. Moreover, action mechanisms could be different depending on the biological activity tested. One hypothesis relies to the fact that these polysaccharides are frequently sulphated, and that they may act as mimetics of glycoaminoglycans (GAG). Glycoaminoglycans constitutes a family of polymers with high sulphate content and are major components of the extracellular matrix in animal tissues. They have been described as antitumor, antiviral, or antioxidant agents in a mechanism directly linked to their sulphate content [69,70,71,72]. In the same way, a linear relationship between the sulphate level and the antiviral activity of EPS from *Porphyridium* against the Herpes virus was proposed [41]. However, the sulphation degree may not be the only parameter to be considered as their position should play a key role [48]. In some cases, presence of uronic acids seems also to be an important parameter, as some polymers exhibiting an anionic behavior (but not sulphated) were found actives [73,74]. Finally, some activities have been described for neutral polysaccharides (no uronic acids nor sulphate groups) and implication of some specific monosaccharides in the structure has been proposed, such as fucose or rhamnose.

Even if some complementary experiments will be necessary to define a more accurate composition and structure, some of the EPS obtained in this study appear thus really promising. Indeed, all present a negative charge, due to a significative content in uronic acids (EPS from *E. coxiae, E. madagascarensis, C. japonica* or *N. cyanea* being the more acidic ones), or a high sulphation level (mainly EPS from Stylonematophyceae, but also from *T. oligopyrenoides* or *N. cyanea*). EPS from *N. cyanea* even exhibit both features, with 37% uronic acids and 24% sulphate groups. Some EPS were found to be weakly sulphated, such as the one from *C. japonica*, however this polymer has shown a quite high rhamnose content, raising hope that it could also be of interest for biological activities. 

## 4. Materials and Methods

### 4.1. Strains and Culture Conditions

#### 4.1.1. Strains

Studied strains belong to Proteorhodophytina subphylum, containing 3 classes: Stylonematophyceae, Rhodellophyceae and Porphyridiophyceae, and were purchased in December 2019 from the Culture Collection of Algae and Protozoa (CCAP, https://www.ccap.ac.uk/, accessed on 1 December 2019). The strains were *Erythrolobus coxiae* CCAP 1393/6, *Erythrolobus madagascarensis* CCAP 1393/3, *Porphyridium sordidum* CCAP 1380/6 and *Timspurckia olygopyrenoides* CCAP 1392/2 from the Porphyridiophyceae subphylum, *Neorhodella cyanea* CCAP 1346/1 and *Corynoplastis japonica* CCAP 1345/1 from the Rhodellophyceae subphylum, *Chroodactylon ornatum* CCAP 1364/1, *Bangiopsis subsimplex* CCAP 1349/1, *Chroothece richteriana* 1353/4, *Rhodaphanes brevistipitata* 1387/1, and *Rhodospora sordida* 1392/1 from the Stylomenatophyceae subphylum. Figure 1 presents a simplified classification of the strains among Rhodophyta phylum.

#### 4.1.2. Cultivation Conditions

All strains were cultivated in F/2 medium [75], modified as previously described [8] (for 1 L in milliQ water: NaCl 28.13 g, KCl 0.77g, CaCl_2_,2H_2_O 1.6 g, MgCl_2_,6H_2_O 4.8 g, NaHCO_3_ 0.11 g, MgSO_4_,7H_2_O 3.5 g, NaNO_3_ 0.598 g and NaH_2_PO_4_,2H_2_O 0.03445 g before to be adjusted to pH 8.0. After sterilization at 120 °C during 20 min, this medium was supplemented with 1 mL of a stock solution of trace elements (for 1 L: Na_2_EDTA 4.16 g, FeCl_3_,6H_2_O 3.15 g, CuSO_4_,5H_2_O 0.01 g, ZnSO_4_,7H_2_O 0.022 g, CoCl_2_,6H_2_O 0.01 g, MnCl_2_,4H_2_O 0.18 g, Na_2_MoO_4_,2H_2_O 0.006 g) and 1 mL of vitamin mix (cyanocobalamin 0.0005 g, thiamine HCl 0.1 g, biotin 0.0005 g) filtered at 0.2 µm. 500mL Erlenmeyer flasks containing 250 mL of medium were inoculated with 15 mL of a subculture. Flasks are maintained in an incubator (Innova 44R, Eppendorf) at 20 °C, 100 rpm mixing, and 60 µmol de photons m^−2^ s^−1^ with a photoperiod of 16:8 h (light:dark).

### 4.2. Analysis during Growth

#### 4.2.1. Biomass

Biomass concentration was assayed during growth by counting on Malassez cells for unicellular strains. In addition, final biomass concentration was evaluated by dry weight measurements for all strains.

#### 4.2.2. Nitrates

Nitrate concentrations were measured on supernatant of culture samples treated by centrifugation (10,000× *g* for 10 min at 20 °C) according to the method of [76] modified by [77]. Briefly, 400 µL of sample (diluted if appropriate) are mixed with 3.6 mL of a 5% perchloric acid solution. Absorbances are read at 210 and 275 nm using a quartz cuvette. NaNO_3_ solutions (between 0.01 and 0.1 g L^−1^) are analyzed following the same protocol, with zero of spectrophotometer carried out at 210 nm for a sample without NaNO_3_, and standard curve established as [NaNO_3_] = f(A_210_). For samples, the absorbance reported on standard curve is corrected using the following equation:

A_sample_ = A_210_ – 2 × (A_275_ – A_275sc_) (1)

With A_210_ and A_275_, raw values of samples at 210 and 275 nm, respectively, and A_275sc_, the mean value of absorbances at 275 nm for the NaNO_3_ solutions of standard curve.

#### 4.2.3. Total Sugar Assay

Total sugars contents in the same supernatants desalted using a 10 kDa membrane were determined according to a modified phenol sulphuric assay [78]. A standard curve was established using glucose between 0.05 and 0.3 g L^−1^. 

### 4.3. Microscopic Observations

Cells were observed under microscope with or without staining. Two kinds of staining were used: periodic acid-schiff staining, that allows to observe presence of polysaccharides [79], and Alcian blue staining, which is specific of carboxylated and/or sulphated polysaccharides [80].

One drop of cellular suspension is put on a microscope slide and air-dried, before fixation with a 4% formalin solution in ethanol. After drying, slides are rinsed 1 minute in tap water. For periodic acid-schiff (PAS) staining, the reagents included in the kit from Sigma-Aldrich (N° 395B) were used. The protocol recommended by the supplier was adapted to fit to our samples, as this staining is generally used for histochemical analysis of tissues. The incubations times were modified as follow. Slides were immersed for 1 min in the periodic acid solution, rinsed several times in distilled water, immersed for 5 minutes in Schiff’s reagent, and rinsed again. Slides were counterstained in hematoxylin solution for 30s, rinsed again, and allowed to air dry before microscopic observation. For Alcian Blue staining, after cells fixation as previously described, slides were immersed for 5 s in a 1% Alcian Blue in 3% acetic acid solution (pH 2.6), rinsed several times in distilled water, and air-dried. Finally, for combined staining, slides were first stained following the periodic acid-schiff procedure, followed by the Alcian Blue one. Microscopic observations were performed using an Olympus CX41 optical microscope (objective ×100), equipped with a Toupcam UA510CA camera (ToupTek). The ToupTek software was used to measure cells sizes and diameters were calculated as the mean of at least 50 cells.

### 4.4. Extraction and Characterization of Polysaccharides 

#### 4.4.1. Extraction and Purification

Soluble extracellular polysaccharides were recovered from culture medium after centrifugation (10,000× *g*, 10 min). Supernatants were diafiltered using vivaflow 200 system (Sartorius) with a 10 kDa NMWCO membrane, in order to remove salts, until a 2 µS cm^−1^ conductivity was reached. After 5 times concentration, polysaccharides solutions were freeze-dried until further analysis. 

Bound exopolysaccharides (BPS) were extracted from cells according to a protocol derived from [29]. Briefly, biomass pellets were resuspended in buffer TAPS (0.05 M), EDTA (0.025 M) and NaCl (0.025 M), heated at 100 °C for 1 h and a second centrifugation was performed (15,000× *g*, 30 min, 20 °C) to recover the cell-free polymer in the supernatant. The cells were re-suspended in the same buffer for a second extraction. The resulting combined supernatants of the two consecutive extractions were precipitated by addition of 10 volumes of 3% cetyltrimethylammonium bromide, which was assumed to allow specific precipitation of acidic polysaccharides. After a new centrifugation step (10,000× *g*, 10 min, 20 °C), pellet was resuspended in 1.0M KCl before precipitation with 3 volumes of 96° ethanol (left overnight at −20 °C) and centrifugation (10,000× *g*, 10 min, 20 °C). These latter solubilization/precipitation steps were repeated 2 times with decreasing KCl concentration solutions (0.75 and 0.3 M) in order to destabilize complex between CTAB and polysaccharide. The last precipitated polysaccharide pellet was then resuspended in milli-Q water. These successive precipitations allowed removing most of the proteins and the other impurities, but the salts were often co-precipitated [81]. In order to achieve greater sample purity, the polysaccharide solution was diafiltered using Vivaflow 200 ultrafiltration system (Sartorius) with a 10 kDa NMWCO membrane in order to remove remaining salts. Diafiltration was stopped when conductivity of filtrate was found as lower than 2 µS cm^−1^. Polysaccharide solution was then concentrated (volume reduction factor of 5) before freeze-drying.

#### 4.4.2. Colorimetric Assays

Purity of extracted polysaccharides was assayed by phenol-sulfuric method as described in Section 4.2.3. Uronic acids and neutral sugar contents of EPS extracts were assayed with meta-hydroxyldiphenyl and resorcinol as described by, respectively [82], and [83] using glucose and glucuronic acid as standards. Quantification of neutral sugars was carried out according to a corrective formula [84]. The results were expressed in mg g^−1^ of d-glucose equivalent (GlcEq) for neutral sugars and in mg g^−1^ of d-glucuronic acid equivalent (GlcAEq) for uronic acids. Sulphur content was determined by the turbidimetric method [85] using K_2_SO_4_ as a standard. Results were expressed in mg g^−1^ of SO_4_ equivalent. Protein content was evaluated by the Lowry method [86] using SAB as a standard.

#### 4.4.3. Monosaccharides Composition

Monosaccharide compositions of polysaccharides were evaluated by High Pressure Anion Exchange Chromatography (HPAEC) on an ICS 3000 (Dionex, Sunnyvale, CA, USA) equipped with pulsed amperometric detection (PAD) and AS 50 autosampler. as previously described [6,9,27,87]. It was assembled with a guard CarboPac PA1-column (4 × 50 mm) and analytical CarboPac PA1-column (4 × 250 mm). Before analysis, polysaccharide samples were hydrolyzed in 2 M HCl for 90 min at 120 °C and neutralized with 2M NH_4_OH. Samples were filtered using 0.2 µm membrane filter and injection volume was fixed at 25 µL. Before each injection, columns were equilibrated by running during 15 min with 18 mM NaOH. Samples were eluted isocratically with 18 mM NaOH for 25 min, followed by a linear gradient between 0 to 0.5 M sodium acetate in 200 mM NaOH for 20 min to elute acidic monosaccharides. Run was followed by 15 min washing with 200 mM NaOH. The eluent flow rate was kept constant at 1 mL min^−1^. Columns were thermostated at 25 °C. Data were collected and analyzed with Dionex Chromeleon 6.80 software (Dionex, Sunnyvale, CA, USA). Constituting monosaccharides were first identified regarding elution time as compared to standards (l-Rha, d-Rib, l-Fuc, l-Ara, d-Xyl, d-Man, d-Gal, d-Glc, d-GalN, d-GlcN, d-GalNac, d-GlcNac, d-GlcA, d-GalA), and then confirmed by the internal standard method (supplementation of a sample by the suspected monosaccharide). Quantification of monosaccharides is then achieved by injecting several concentrations of standard monosaccharides and plotting response area as function of concentration.

#### 4.4.4. Fourier Transform Infrared Spectroscopy

Fourier-Transform Infrared (FT-IR) measurements were carried out using a VERTEX 70 FT-IR instrument. Dried polysaccharide was dispersed on ATR A225 diamond. The IR spectra (50 scans. 4 cm^−1^ resolution) were recorded at room temperature (referenced against air) in the wavenumber range of 500–4000 cm^−1^. Spectra were analyzed with OPUS 7.2 software (Bruker Optics, Ettlingen, Germany).

## Figures and Tables

**Table 1:** Growth and EPS parameters.

STRAINS	EPS Production Phase	Apparent µmax (d^−1^)	Dt (d)	Final Biomass Production (×10^6^ cell·mL^−1^)	Final Biomass Production (g L^−1^)	Final RPS Production (g L^−1^)	Productivity RPS (g EPS/g DW Biomass)	Productivity RPS (mg EPS/10^6^ cell)	Productivity BPS (g EPS/g DW Biomass)
*T. oligopyrenoides*	Stationary	0.152	4.55	31.8	2.2	1.12	0.35	0.035	nd
*E. coxiae*	End of Log/ Stationary	0.2632	2.63	28.34	2.04	1.43	0.47	0.05	nd
*E. madagascarensis*	End of Log/ Stationary	0.1026	6.75	20.9	1.79	1.63	0.78	0.078	nd
*P. sordidum*	Log/ Stationary	0.151	4.58	28.2	1.81	1.91	0.68	0.068	nd
*N. cyanea*	End of Log/ Stationary	0.172	4.02	15.4	2.35	2.32	0.99	0.151	nd
*C. japonica*	Log/ Stationary	0.067	10.34	6	2.49	1.59	0.64	0.265	nd
*C. ornatum*	nd	nd	nd	nd	1.2	0.014	0.0086	nd	0.24
*C. richteriana*	nd	nd	nd	nd	0.64	0.023	0.0018	nd	0.19
*B. subsimplex*	nd	nd	nd	nd	0.46	0.011	0.0013	nd	0.08
*R. sordida*	nd	nd	nd	nd	0.72	0.021	0.0038	nd	0.14
*R. brevistipitata*	nd	nd	nd	nd	0.51	0.013	0.0021	nd	0.11

RPS: Released polysaccharide (EPS free in the culture medium); BPS: Bound polysaccharide (EPS bound to the cells and extracted as described in Materials and Methods section). Final biomass (g L^−1^) was determined by dry weight measurement after centrifugation and recovery of the whole biomass. Final EPS concentration (g L^−1^) is obtained after extraction and purification of the supernatant, and corrected by the purity of the extract, determined by total sugar assay. Dt: doubling time, nd: not determined.

**Table 2:** FT-IR characteristic peaks and assignments. Letters (A to V) refer to bands shown on Figure 5.

	Wavenumber (cm^−1^)	Assignment	Reference	*Ps*	*Ec*	*Em*	*To*	*Nc*	*Cj*	*Co*	*Rb*	*Bs*	*Rs*	*Cr*
A	3200–3400	OH stretching	[30]	3309	3305	3306	3350	3358	3290	3301	3329	3318	3340	3303
B	2950–2960	CH_3_ stretching	[30]	nd	nd	nd	nd	nd	nd	nd	nd	2954	2954	2953
C	2920–2930	CH_2_ stretching	[30]	2923	2927	2927	2928	2924	2923	2923	2925	2923	2925	2924
D	2870–2880	CH_3_ stretching	[30]	nd	nd	nd	nd	nd	nd	2873	2873	2871	2875	2875
E	2845–2855	CH_2_ stretching	[30]	2855	nd	nd	nd	2845	2852	2852	2848	2854	2854	2854
F	1725–1740	*O*-Ac	[38]	1730	nd	1725	1726	1730	1726	nd	nd	nd	nd	nd
G	1630–1660	C=O	[47]	1640	1640	1638	1650	1641	1638	1648	1641	1638	1633	1639
H	1585–1605	COO^−^ stretching	[32,33,34]	1599	1596	1597	1605	1605	1599	nd	nd	nd	nd	nd
I	1525–1540	amide II (proteins)	[30]	1536	1536	1537	1535	1536	1536	1542	1531	1535	1536	1535
J	1455–1470	CH_3_ bending	[30]	1460	1461	1461	1458	1461	1461	1459	nd	nd	nd	1460
K	1400–1420	COO^−^ stretching	[31,32,33]	1414	1414	1414	1413	1415	1413	1412	1409	1413	1414	1414
L	1340–1350	CH bending	[30]	1352	1354	1355	1355	1358	1353	1346	1353	1354	1353	1348
M	1245–1255	S=O	[36,37]	1242	1246	1251	1245	1247	1247	1252	1252	1255	1257	1257
N	1240–1250	*O*-Ac	[9]											
O	1215–1225	S=O	[35]	1211	nd	nd	1213	1216	nd	1221	1221	1217	1217	1215
P	1135–1145	C-O-C	[31]	1143	1145	1143	1138	1144	1146	1149	1152	1144	1143	1142
Q	1165–1075	C-OH	[31]	1068	1076	1074	1075	1068	1080	1075	1072	1075	1068	1071
R	1020–1030	C-C	[31]	1031	1030	1031	1025	1020	1036	1025	1023	1030	1025	1020
S	980–985	C-H	[30]	980	975	977	977	979	980	985	nd	975	nd	nd
T	925–935	C-O-C (AnGal)	[45]	nd	nd	nd	923	nd	nd	933	932	934	nd	nd
U	890–900	β linkage	[46]	897	894	894	nd	898	895	nd	891	nd	nd	nd
V	840–850	α linkage	[46]	nd	nd	nd	845	nd	nd	850	847	843	851	852

nd: peak can’t be detected and assigned. It can be present but masked by another closed peak. Ps: *Porphyridium sordidum*, Ec: *Erythrolobus coxiae*, Em: *Erythrolobus madagascarensis*, To: *Timspurckia oligopyrenoides*, Nc: *Neorhodella cyanea*, Cj: *Corynoplastis japonica*, Co: *Chroodactylum ornatum*, Rb: *Rhodaphanes brevistipitata*, Bs: *Bangiopsis subsimplex*, Rs: *Rhodospora sordida*, Cr: *Chroothece richteriana*.

**Table 3:** Global composition of samples (Total carbohydrates and proteins), and exopolysaccharides (Neutral sugars, Uronic acids, and Sulphate content).

Strains	Samples Composition [% Mass]		EPS Composition [% Mass (g / 100g EPS)]		
	% Total Carbohydrates	% Proteins	% Neutral Sugars	% Uronic Acids	Sulphate Groups (%, eq. SO_4_)
*E. coxiae*	62	5.1	68.2	30.2	1.6
*E. madagascarensis*	68.1	5.7	67.1	31	1.9
*P. sordidum*	61.5	9.5	72.4	20.8	6.8
*T. oligopyrenoides*	63.6	6.4	67.5	18.1	14.4
*C. japonica*	78.4	11	61.3	37.5	1.2
*N. cyanea*	71.5	8.6	38.7	37.1	24.2
*C. ornatum*	64.3	5.3	69.1	11	19.9
*C. richteriana*	57.2	8.7	61.2	12.4	26.4
*B. subsimplex*	45.8	11.4	71.4	9.5	19.1
*R. sordida*	47.3	12.3	66.8	4.5	28.7
*R. brevistipitata*	51.2	9.4	79.7	2	18.3

**Table 4:** Monosaccharide compositions of EPS from microalgae belonging to Porphyridiophyceae class.

% Molar Ratio	*E. coxiae*	*E. madagascarensis*	*T. oligopyrenoides*	*P. sordidum*	*P. sordidum*	*P. purpureum*	*P. marinum*	*F. sanguinaria*
Fucose	4.50%	1.90%	1.70%	0.00%	0.00%	0.00%	1.00%	0.00%
Rhamnose	0.40%	0.50%	0.00%	0.30%	0.00%	0.00%	0.00%	10.00%
Arabinose	2.20%	2.80%	0.20%	0.00%	0.00%	0.00%	0.00%	2.00%
Galactose	12.00%	22.30%	28.20%	21.10%	33.00%	32.00%	28.00%	21.00%
Glucose	1.00%	1.70%	1.50%	12.80%	23.00%	21.00%	18.00%	6.00%
Xylose	58.30%	49.10%	51.00%	46.40%	39.00%	41.00%	47.00%	47.00%
Galacturonic acid	0.60%	0.50%	0.20%	1.10%	0.00%	0.00%	0.00%	0.00%
Glucuronic acid	20.90%	21.10%	18.00%	18.30%	5.00%	4.00%	6.00%	14.00%
References	This study				[24]	[24]	[6]	[9]

First major monosaccharide2nd major monosaccharide3rd major monosaccharide

**Table 5:** Monosaccharide compositions of EPS from microalgae belonging to Rhodellophyceae class.

% Molar Ratio	*Corynoplastis japonica*	*Neorhodella cyanea*	*Rhodella violacea*	*Rhodella maculata*
Fucose	0.00%	0.00%	0.00%	0.00%
Rhamnose	14.10%	0.20%	2.00%	5.00%
Arabinose	0.00%	0.00%	1.00%	2.00%
Galactose	9.10%	10.60%	52.00%	45.00%
Glucose	0.80%	1.70%	7.00%	1.00%
Xylose	50.10%	62.20%	34.00%	42.00%
Galacturonic acid	0.00%	0.00%	0.00%	0.00%
Glucuronic acid	25.90%	25.40%	3.00%	5.00%
References	This study		[7]	[47]

First major monosaccharide2nd major monosaccharide3rd major monosaccharide

**Table 6:** Monosaccharide compositions of EPS from microalgae belonging to Stylonematophyceae class.

% Molar Ratio	*Chroodactylum ornatum*	*Chroothece richteriana*	*Bangiopsis subsimplex*	*Rhodaphanes brevistipitata*	*Rhodospora sordida*
	BPS	BPS	BPS	BPS	BPS
Fucose	9.90%	0.60%	0.20%	0.00%	0.60%
Rhamnose	4.50%	0.40%	0.20%	0.80%	0.30%
Arabinose	2.00%	0.00%	0.30%	0.40%	1.00%
Galactose	19.30%	58.40%	78.20%	86.80%	31.20%
Glucose	30.10%	6.30%	5.40%	3.30%	6.40%
Xylose	19.80%	27.50%	5.60%	6.70%	58.70%
Galacturonic acid	5.80%	1.50%	0.80%	0.00%	0.70%
Glucuronic acid	6.40%	5.50%	9.30%	2.00%	1.70%

First major monosaccharide2nd major monosaccharide3rd major monosaccharide

## Supplementary Materials

The following supporting information can be downloaded at: https://www.mdpi.com/article/10.3390/md20040246/s1, Figure S1: HPAEC-PAD chromatograms and assignments of peaks for standards (A, standards injected in several mixes to avoid quantification errors due to overlapping), and EPS from *Erythrolobus coxiae* (B), *Erythrolobus madagascarensis* (C), *Timspurckia oligopyrenoides* (D), *Porphyridium sordidum* (E), *Neorhodella cyanea* (F), *Corynoplastis japonica* (G), *Chroodactylon ornatum* (H), *Chroothece richteriana* (I), *Bangiopsis subsimplex* (J), *Rhodaphanes brevistipitata* (K), and *Rhodospora sordida* (L).
