# Supplementary material for: Exploring the Diversity of Red Microalgae for Exopolysaccharide Production
Source: Mar Drugs. 2022 Mar 31;20(4):246. doi: 10.3390/md20040246 (PMC9031348; doi:10.3390/md20040246)
Supplement: Supplementary file 1 [file marinedrugs-20-00246-s001.zip › marinedrugs-1644442-supplementary.pdf]

Supplementary Figure S1: HPAEC-PAD chromatograms and assignments of peaks for standards (A, standards injected in several mixes to avoid quantification errors due to overlapping), and EPS from *Erythrolobus coxiae* (B), *Erythrolobus madagascarensis* (C), *Timspurckia oligopyrenoides* (D), *Porphyridium sordidum* (E), *Neorhodella cyanea* (F), *Corynoplastis japonica* (G), *Chroodactylon ornatum* (H), *Chroothece richteriana* (I), *Bangiopsis subsimplex* (J), *Rhodaphanes brevistipitata* (K), and *Rhodospora sordida* (L).

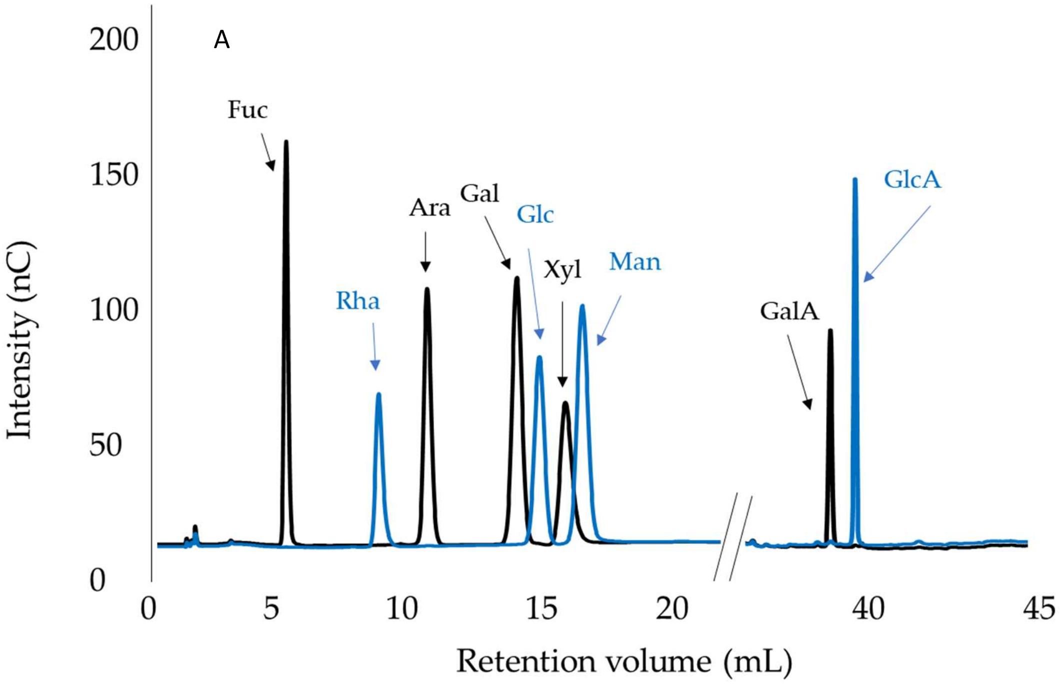

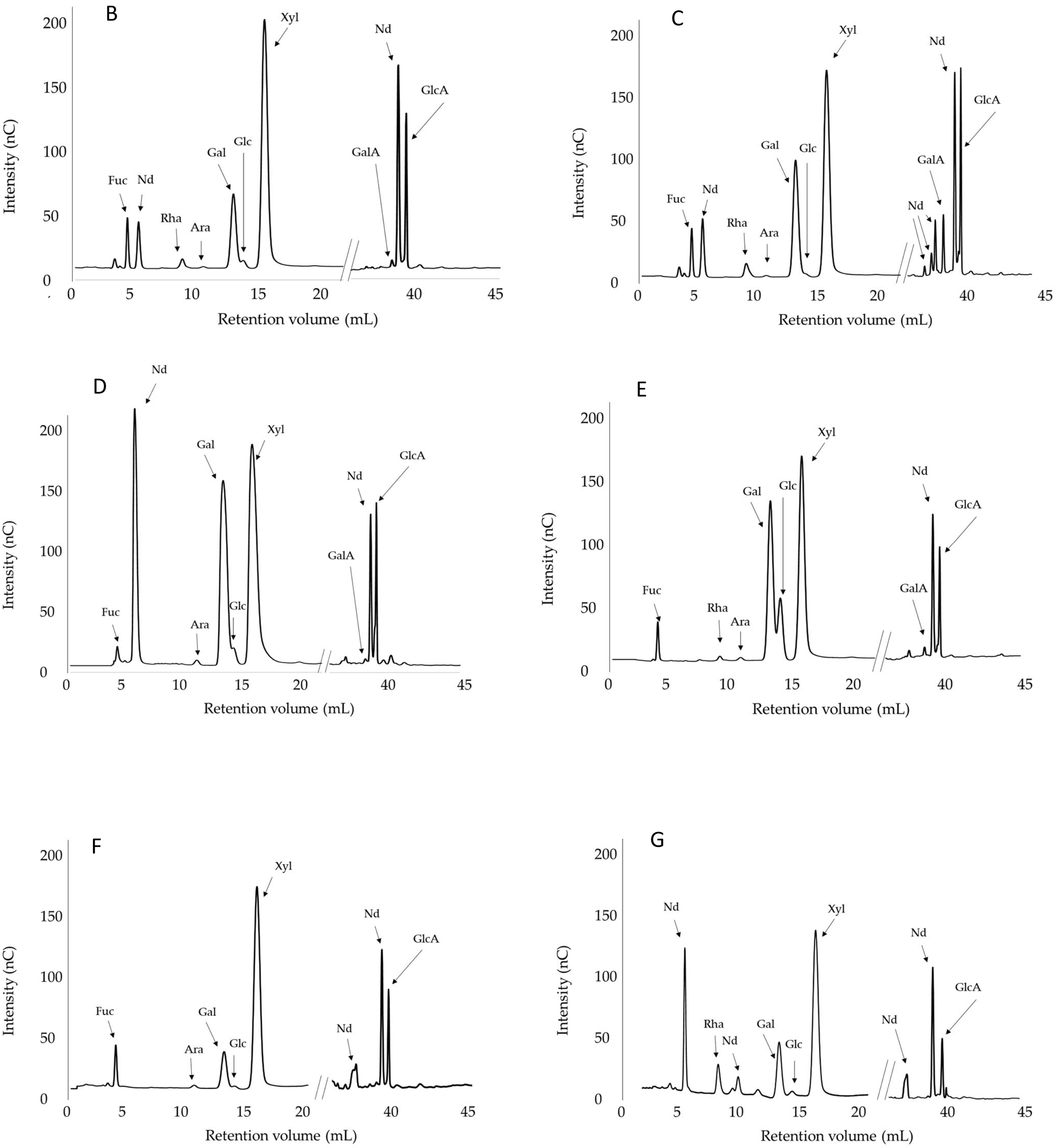
B
C
D
E
F
G
Intensity (nC)
Retention volume (mL)
Fuc
Nd
Rha
Ara
Gal
Glc
Xyl
GalA
GlcA
0
50
100
150
200
0 5 10 15 20 40 45

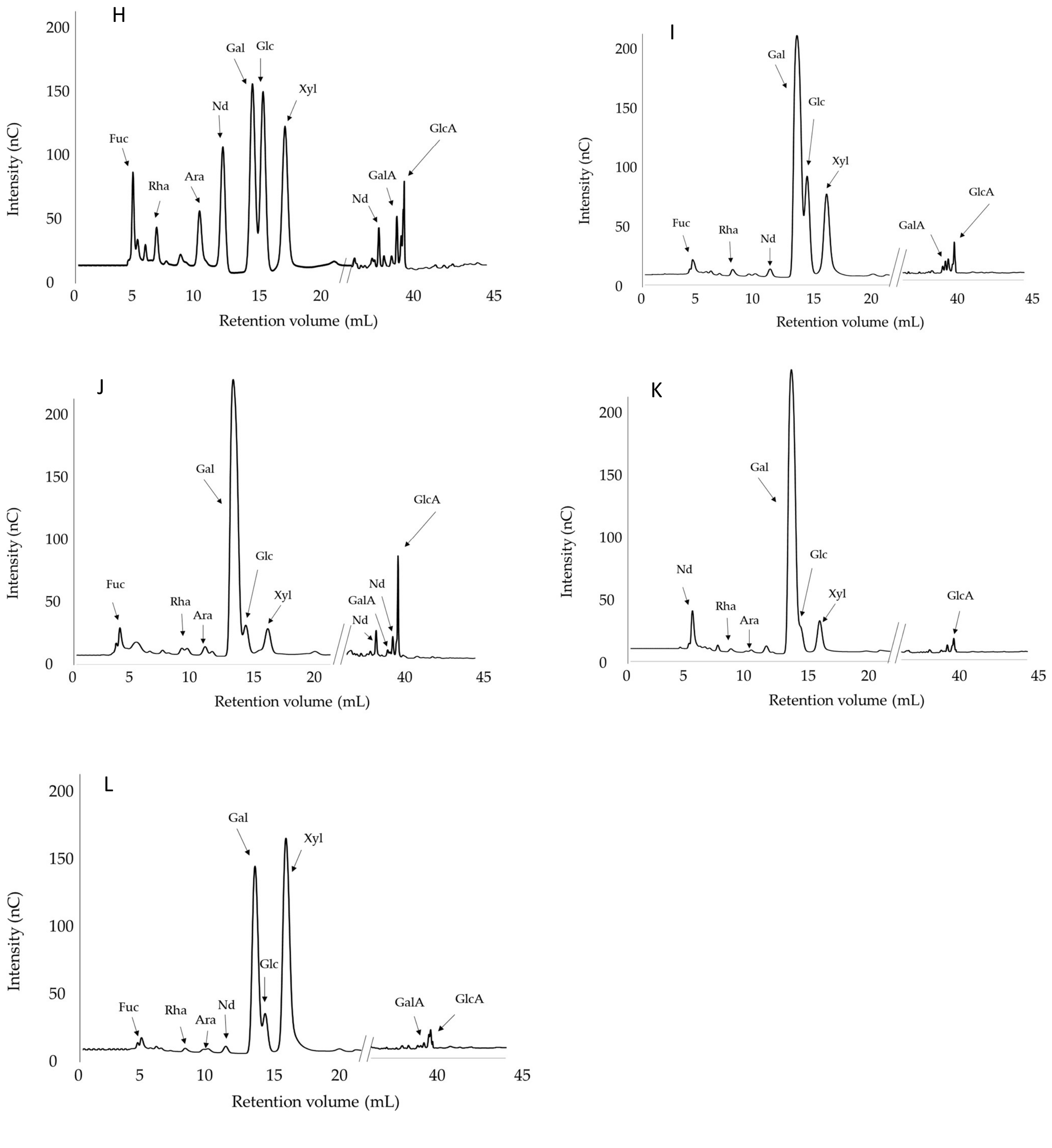
H
I
J
K
L
Intensity (nC)
Retention volume (mL)
Fuc
Rha
Ara
Nd
Gal
Glc
Xyl
GalA
GlcA
